# Exploring extramedullary hematopoiesis: unraveling the hematopoietic microenvironments

**DOI:** 10.3389/frhem.2024.1371823

**Published:** 2024-05-28

**Authors:** Guadalupe Rivera-Torruco, Marcus O. Muench, Ricardo Valle-Rios

**Affiliations:** 1 Cell Therapy Core, Vitalant Research Institute, San Francisco, CA, United States,; 2 Department of Laboratory Medicine, Medical Center, University of California, San Francisco, San Francisco, CA, United States,; 3 Research Division, Faculty of Medicine, National Autonomous University of Mexico, Mexico City, Mexico,; 4 Laboratorio de Investigación en Inmunología y Proteómica, Hospital Infantil de México Federico Gómez, Mexico City, Mexico

**Keywords:** hematopoiesis, stem cell, embryonic development, hematopoietic progenitor cells, extramedullary hematopoiesis

## Abstract

Hematopoiesis is a process by which all blood cells are formed. The mechanisms controlling it have been studied for decades. Surprisingly, while hematopoietic stem cells are among the most extensively studied stem cell types, the complete understanding of how they are regulated during development, adulthood, or in non-homeostatic conditions remains elusive. In this review, our primary focus is on research findings that explore where hematopoietic precursors are found in adults outside their primary niches in the bone marrow. This phenomenon is termed extramedullary hematopoiesis (EMH). Early in development hematopoietic stem cells migrate through different regions within and outside the embryo and later the fetus. Although, the primary home for hematopoietic progenitors is the adult bone marrow, it is now recognized that other adult organs may act as hematopoietic progenitor reservoirs both in mice and humans. The first reports about this topic were principally originated from clinical observations, in cases where the bone marrow was malfunctioning, leading to an aberrant hematopoiesis outside the bone marrow. It is worth highlighting that those extramedullary organs, like the small intestine or fat tissue, contain subsets of fully functioning hematopoietic progenitors demonstrated by both *in vitro* and *in vivo* studies. Nonetheless, there are still some unanswered questions regarding the source of these cells, how they differ in function compared to their counterparts in the bone marrow, and the specific roles they play within the tissues where they are located.

## 1 Introduction

Stem cells are a unique cell type with the remarkable ability to serve as building blocks for all cell types in the body. The studies of Till and McCulloch were instrumental in shaping our understanding of stem cells, and their work continues to have a lasting impact on both basic research and clinical applications in the field of regenerative medicine and beyond McCulloch and Till (1). Over the past half-century, researchers have discovered combinations of physical properties, cell-surface markers, and devised assays to measure stem cell function that allow for the identification and separation of stem cell populations in adult tissues like the skin, intestinal epithelium, brain, and the hematopoietic system.

The best characterized stem cells are the hematopoietic stem cells (HSCs), which reside primarily within the bone marrow (BM) during adulthood. Murine HSCs are distinguished by the absence of cell-surface markers present on lineage (Lin)-committed hematopoietic cells. Additionally, these cells express high levels of the stem cell factor receptor (c-kit, CD117) (2) and stem-cell antigen 1 (Sca1) (3, 4); this Lin^−^c-kit^+^Sca1^+^ subset is commonly referred to as LSK cells (4, 5).

HSCs account for 0.00125–0.00425% (12–42 cells per million) of whole BM in mice (6). These HSCs are comprised of two main types: Long-term HSCs (LT-HSCs) or dormant HSCs and short-term HSCs (ST-HSCs) or activated HSCs. These populations of HSCs can be differentiated by expression of the anti-adhesive sialomucin CD34 (7); mouse LT-HSCs do not express CD34 until they become activated ST-HSCs (6, 8, 9). Other methods of HSC isolation often begin with enrichment of LSK cells, a heterogeneous population of hematopoietic precursors, with only a small subset consisting of LT-HSCs. According to flow cytometry analysis, the frequency of LT-HSCs within the LSK population is approximately 10% (10, 11). However, *in vivo* analyses may vary in the frequencies of long-term reconstituting cells measured among LSK cells: frequencies as low as 2% (12) or as high as 20% (13) having been reported. Frequency variation in the BM progenitor pool may be related to mouse age (14), sex (15), and strain variation (16, 17). For example, the frequency of LT-HSCs cells increases with age (10). In addition to CD34, other markers of HSCs that have been used to differentiate LSK cells are the SLAM family receptors (CD150 and CD48) (13), CD90 (5), and the capacity of HSCs to remove intravital dyes like Rhodamine-123 (3, 18).

In humans, the HSC population, similar to mice, is characterized by the absence of maturity markers and the expression of CD34 (Lin^−^CD34^+^) (19). Furthermore, HSCs are found to be more abundant among CD34^+^ cells expressing CD4 (20), CD90 (21), CD133 (22), and EPCR (23), while lacking expression of CD38 (24–26), CD45RA (27), and CD71 (28). Similar to murine HSCs, there exists a rare subset of HSCs characterized by the absence of an extensive lineage marker panel and CD34 (29). These CD34^−^ HSCs have been identified in human cord blood and are known to express GPI-80 (30), CD90 (31), CD93 (31), and CD133 (32). While transplant success using CD34 selection suggests the presence of functional HSCs within the CD34^+^ fractions of neonatal and adult cells, experimental evidence indicates that pre-natal CD34^−^ HSCs may be more primitive than their CD34^+^ counterparts (31, 33).

## 2 Hematopoiesis: from development to adulthood

During embryonic development, the earliest hematopoietic progenitor cells emerge in the yolk sac, appearing around day 17 in humans and embryonic day 7.5 (E7.5) in mice (34). These progenitors, originating from the mesodermal layer, form clusters known as “blood islands” within the yolk sac, closely associated with endothelial and hematopoietic cells. Human yolk sac development progresses through three phases: 1) a formative period, 2) a functional period, and 3) a period of regression. During the functional phase, which typically occurs during the second stage, the yolk sac comprises cells of both mesodermal and endodermal origin, housing endothelial and early hematopoietic cells (35). In humans, at 3–4 post-conception week (PCWs), the first generation of hematopoietic cells proliferate in the yolk sac and extraembryonic mesenchyme, primarily consisting of “primitive” erythroblasts (megaloblasts) which are present in circulating blood from 4 PCWs onward (36). Shortly after the initiation of blood island formation and primitive hematopoietic progenitor generation, endothelial cells within the para-aortic splanchnopleura region begin producing definitive HSCs. In the mouse, this process typically takes place around E9.5 (37), while in humans, it occurs briefly around Carnegie Stages (CS) 9–12 which is equivalent to 19–27 days of embryonic development (38). Subsequently, the para-aortic splanchnopleura region gives rise to most tissues in the aorta-gonad-mesonephros (AGM) (37–39), which emerges approximately after E10.5 in mouse and after the 27th day of pregnancy in humans as an active site of hematopoiesis (38, 40–42).

In mice, HSCs are present in the placenta at the same time as they are in the AGM region (43). Similarly, hematopoietic progenitors are present in the human placenta at approximately 5–6 PCWs (CS 14–16) (44–46). Human HSCs capable of long-term multilineage engraftment in immunodeficient mice were detected from placenta as early as 6 weeks of gestation (CS 17) (45). The human chorion, an extra-embryonic tissue that shares a developmental origin with placenta but is much less vascularized, also contains hematopoietic precursors and HSCs at 5–6 weeks of gestation capable of long-term reconstitution (46, 47). Hematopoietic progenitors in the early stages of placental/chorionic development may originate from extra-embryonic sources. A recent study demonstrated that primitive erythromyeloid progenitors in the placenta are distinct from definitive hematopoietic stem cells due to their absence of HLF expression and their limited lineage commitment primarily to the erythromyeloid lineage (48).

As embryonic development progresses, the liver becomes another important hematopoietic niche. Intraembryonic HSCs are located within the fetal liver of rodents (49) and humans (50) shortly after the liver has formed. It is known that HSCs migrate from the AGM to fetal liver, although the exact migration pathways are still not fully understood (51) it is known to be guided in response to stromal CXCL12 (52).

In mice, the liver begins to form as a diverticulum from the floor of the embryonic gut around E9 (53). Subsequently, the murine liver bud experiences accelerated growth as it becomes vascularized (54). It is believed that primitive progenitors from the yolk sac migrate to the fetal liver through the vitelline vein around E11 (55). By this stage, the murine liver harbors primitive erythromyeloid progenitors derived from the yolk sac (56). Additionally, AGM-derived HSCs begin to populate the murine fetal liver starting at E11, coinciding with maximal AGM activity (57). Subsequently, fetal liver cells at E12 undergo a significant expansion, up to 38-fold, and exhibit long-term reconstitution capability when transplanted into an irradiated mouse, indicating the presence of definitive HSCs by E12, as opposed to E10 and E11 cells, which did not result in reconstitution (58). This observation aligns with the decrease in HSCs observed in the AGM region by E12 (57).

An early and noteworthy investigation illustrated various hematopoietic cell lineages within the human embryonic liver as early as 5 PCW (CS 14–15), delineating their morphological characteristics (59). However, the cells may have comprised circulating primitive erythro-myeloid progenitors and their progeny, as they do not express CD34 and have been observed as early as 23 days of development (CS 10) (50). Recent studies indicate that the liver is initially populated by molecularly defined HSCs around 6 PCWs (CS 16) (60). Although CD34^+^ progenitors are detected in the human fetal liver as early as 30 days of development (CS 13) (50), several investigations indicate that transplantable HSCs are first present in the embryonic liver shortly thereafter, at 5–6 PCWs (CS 14–16) (61–64).

From this time forward until midgestation, the human liver is the primary intra-embryonic hematopoietic organ (65). Erythropoiesis dominates the hematopoietic output of the liver in order to meet the demands of an expanding blood volume (66, 67). Indeed, flow cytometric analysis of whole fetal liver indicate that CD235a *in vitro* erythroid cells are the dominant cell type in the liver during midgestation (68). In addition to erythropoiesis, liver hematopoiesis includes elements of myeloid and lymphoid precursors (26, 69–71). Single-cell transcriptome profiling of human fetal liver further confirmed the presence of HSCs and MPPs with the capability to generate functional innate lymphoid cells, T-, and B-lymphocytes, natural killer (NK), and CD34^+^ myeloid progenitors (72).

Interestingly, some long-lived myeloid cells are found in adult tissues like the lungs or peritoneal cavity. These so-called “tissue resident macrophages” emerge from the yolk-sac instead of progenitors derived from the AGM Gomez (56) or fetal liver (73). Also, some lymphoid populations like the B-1 B cells originate in the yolk-sac, which represent part of the humoral immune response but not the classic adaptive immune system. Similarly to tissue resident macrophages, this subset of B cells are rarely found circulating in the blood stream (74), and undergo self-renewal in the periphery (75). It is noteworthy that the nature of these yolk-sac derived leukocytes have more in common with innate immunity than adaptive immunity as reviewed in (76). While the presence of B-1 cells in humans has been controversial (74).However, a recent comprehensive comparative single-cell sequencing analysis of human fetal and adult tissues has presented compelling evidence, according to these authors, supporting the origin of human fetal B-1 cells (77).

Finally, the definitive hematopoietic organ, the BM, becomes hematopoietic at E15.5 in mouse (78) and at 10.5 PCW in humans (79). Although the first HPSCs in the murine bone marrow are found as early as E15.5 and E16 (80) the amount of LT-HSCs found a these stages is limited and steadily increasing until birth (78). The mouse BM is seeded by HSCs originating in the fetal liver (81). In humans fetal BM is also colonized by circulating hematopoietic precursors derived from fetal liver found in relative abundance during early gestational periods compared to neonates (82, 83). Human fetal BM is first found in the long bones of the fetus as these bones are the first to become large enough to contain a marrow. A recent investigation revealed that although HSCs with defined phenotypes are present in the human BM at 10 PCW, they attain full functionality only after reaching 12 PCWs (84). The BM becomes the dominant site of hematopoiesis early in the second half of gestation as the marrow increases in size relative to the liver.

**FIGURE 1:**
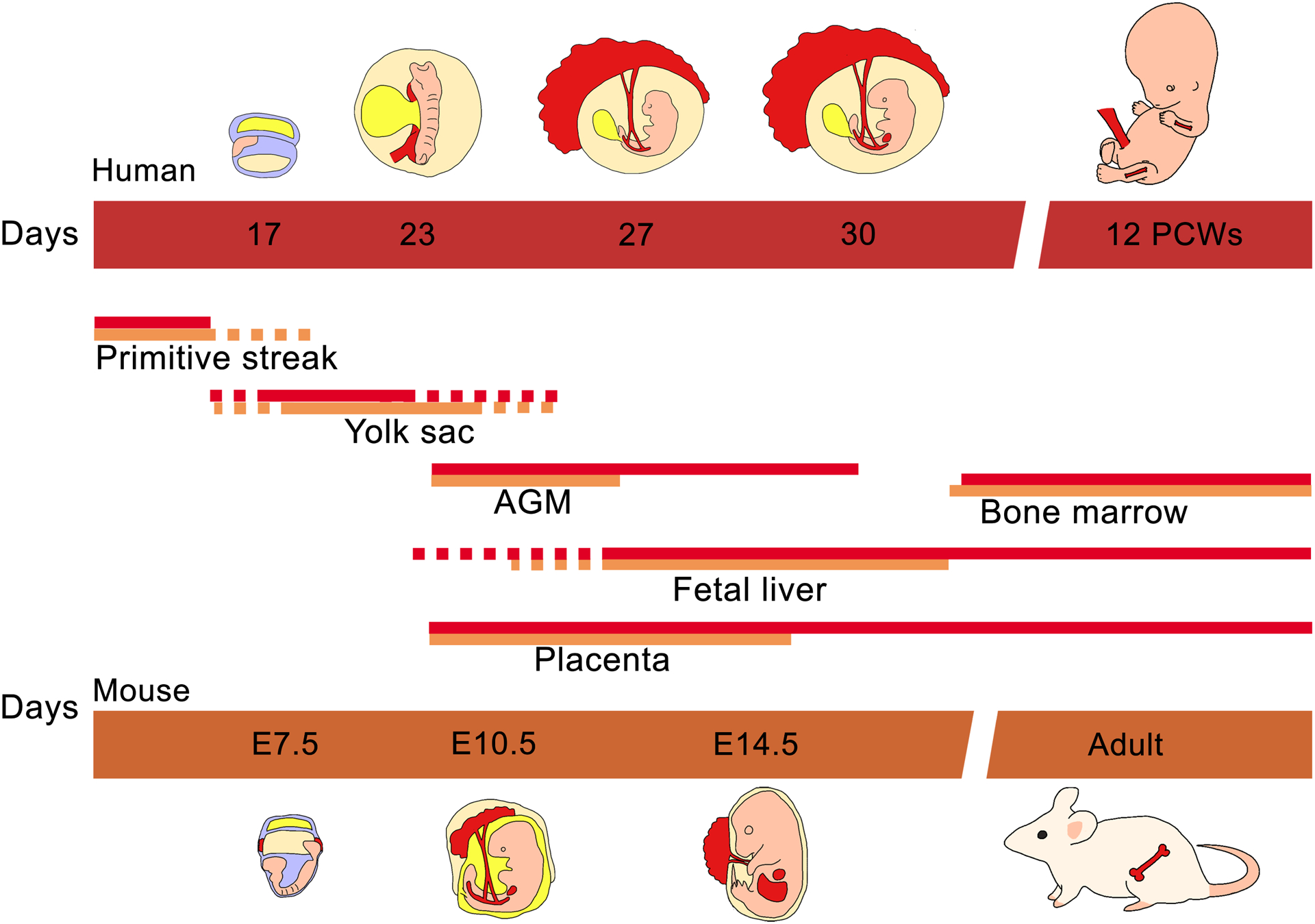
Ontogeny of hematopoiesis in humans and mice. The early hematopoietic progenitor cells emerged in the primitive yolk sac of both human and mouse. While the embryo continues its development, the mesoderm gives rise to vascular structures like the AGM where the definitive hematopoietic cells are produced. Almost at the same time the placenta and embryonic liver are colonized by AGM progenitors. Finally fetal liver hematopoietic cells migrate to populate the bone marrow.

It is worth mention that the fetal spleen is an important hematopoietic tissue in mice bridging the peri-natal period when hematopoiesis diminishes in the liver and before the BM forms. Humans and mice differ in this regard as the spleen contains but an insignificant number of hematopoietic precursors at any time during gestation (85, 86). Figure 1 illustrates the temporal migration of HSCs in human and mouse as well as the tissues where hematopoiesis is normally found.

## 3 Adult extramedullary hematopoiesis

EMH can occur in response to both homeostatic and pathologic conditions, such as acute infection or inflammation. Organs like the spleen and liver can start producing blood cells due to local production of hematopoietic growth factors like GM-CSF, TNFα, and IL-3 (87, 88). In chronic pathologies, the BM may lose its ability to function effectively, leading to increased reliance on organs like the liver, spleen, or even the lungs for blood cell production. In mice, splenic EMH readily occur when the capacity of the BM to produce blood cells is exceeded, recalling the significant role the spleen plays during the perinatal period in hematopoietic development. However, large mammals possessing substantial bone marrow reserves may not demonstrate a comparable propensity to transition blood cell production from the bone marrow to the spleen.

Myelofibrosis, diffuse osseous metastatic disease, leukemia, sickle cell disease, and thalassemia are among the primary factors contributing to human extramedullary hematopoiesis. (89). Many reports have found the presence of hematopoietic progenitors in extramedullary organs due to defective hematopoiesis in the BM due to any of these diseases (Table 1). Interestingly, some cases of EMH are found after inflammatory conditions such as inflamed joints (115), or lung infection (116, 117). Table 1 summarizes reports were EMH is found secondary to disease.

Pathological EMH is a secondary problem diagnosed principally with radiological studies, computed tomography (CT) or magnetic resonance imaging (MRI) (Table 1). Following resection, the anomalous mass is examined by pathologists, the most common techniques used to determine a hematological signature include staining for glycophorin (GPA, CD235a) for erythroid progenitors and Giemsa (118) or H&E (Hematoxylin and Eosin) staining for morphology (89). Sometimes immunohistochemistry (IHC) (104, 106, 111) or flow cytometry (FC) (98) with antibodies against known progenitor and mature immune cells or colloidal scans (93, 105) are used.

Although limited, there are some reports showing that HSPCs are present in tissues outside the BM under homeostatic conditions both in human and animal models. Furthermore, it has been established that these cells exhibit re-populating properties similar to those found in the BM. For example, tissues like the small intestine in humans contain hematopoietic progenitors expressing CD34 and CD45 (119). Later work showed not only a distinct phenotype but also confirmed the presence of hematopoietic colony-forming cells using clonal analysis *in vitro* (CFU-c). Additionally, it demonstrated the potential for long-term chimerism in individuals receiving intestinal allografts (120).

Another potential host for hematopoietic progenitors in humans is the stromal-vascular fraction (SVF) of adipose tissue. In pathological conditions such as chronic myeloid leukemia, adipose tissue has the potential to transform into a hematopoietic niche. In this context, leukemic cells express a fatty acid transporter to enhance their communication with the adipose microenvironment and evade chemotherapy (121). Under homeostatic conditions, this tissue harbors a subset highly enriched in CD34^+^ and VEGFR2^+^ (KDR) cells with absent CD45 expression. Interestingly, despite the lack of CD45 expression, these cells were able to originate hematopoietic colonies in a CFU-c assay (122). Notably, murine SVF within adipose tissue contains similar progenitors defined by a LSK phenotype and are capable of long-term multi-lineage reconstitution when transplanted into a recipient (123).

Among other organs known to exhibit pathology associated EMH, the lungs are included. While there is no conclusive evidence confirming the presence of HSCs in human lungs, there is evidence supporting their existence in mice. Initial observations in mouse lungs have revealed the occurrence of a cell subset, VEGFR2^+^CD31^+^ cells, which can produce endothelial cells, smooth muscle cells and form hematopoietic colonies *in vitro* (124). Anatomical descriptions of lung CD34^+^Sca1^+^-kit^+^ cells showed these cells are located in close proximity to blood vessels, and possibly belong to a unknown subset of hematopoietic progenitors (125). Furthermore it has been reported that mouse lungs are active hematopoietic organs, indeed they account for 50% of platelet production and are a source of hematopoietic progenitor and HSCs with a phenotype and repopulating activity similar to its counterparts in the murine BM (116).

Since the lungs are not classically known to be hematopoietic niches, it has been hypothesized that lung vasculature may function as an adaptive niche for HSCs, principally ST-HSCs, since LT-HSCs were not found by Lefrançais et al. in the lungs (116, 126). The lungs harbor more than 50 different cell types (127), the most likely subset to be nurturing HSCs are pericytes or distinct blood vessels, further work is needed to elucidate this question.

Besides the lungs, reports have indicated that the liver and spleen also display EMH associated with pathologies. Over the last decade, numerous studies have revealed that both organs harbor functional hematopoietic progenitors along with the necessary microenvironment to sustain them. A recent report has indicated the presence of an LSK population in the mouse spleen, along with its ability to expand when the *Txl1* gene is overexpressed in splenic stromal cells (128). As for hepatic hematopoiesis, there is evidence that supports that liver sinusoid endothelial cells provide an appropriate hematopoietic environment that supports the proliferation and differentiation of hematopoietic progenitors in the mouse (129). Moreover, adult murine liver was shown to support human HSC engraftment, showing long-term multilineage hematopoiesis (130). In addition, donor chimerism after liver transplantation is a common finding, this comes from having a significant amount of donor-derived hematopoietic cells in the liver graft (131, 132). Liver-derived hematopoietic engraftment has even been documented to lead to a complete blood group change in the recipient (133).

**FIGURE 2:**
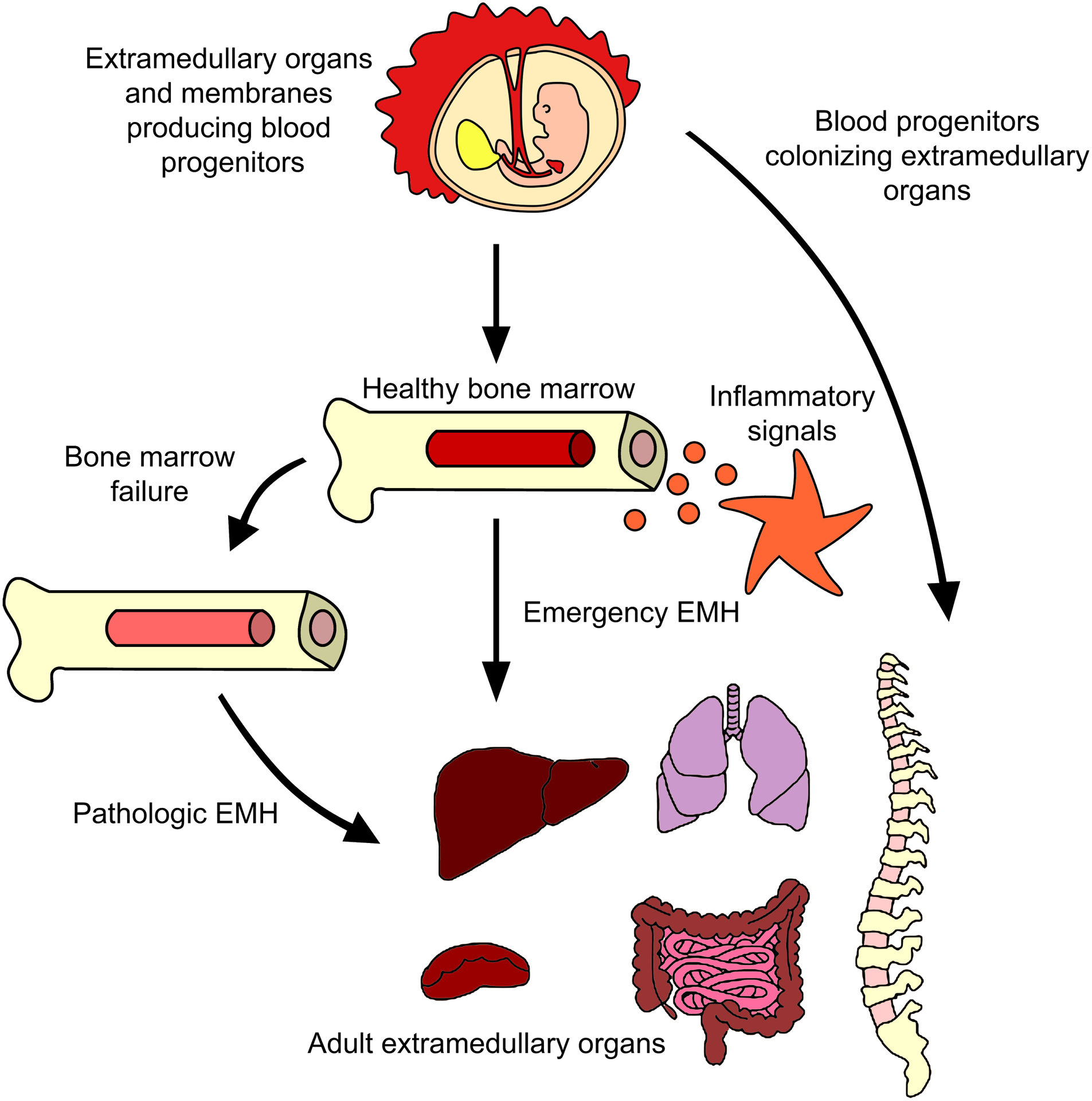
Extramedullary hematopoiesis in adults. Many reports have described the generation of progenitor cells outside the BM in adults. The major evidence points to BM failure and inflammation being the causes of some blood progenitors to emigrate from BM to organs like liver or spleen, although evidence of lungs or paraspinal EMH have been described. Murine models showed the presence of functional hematopoietic precursos in those tissues even without a disease or inflammatory insult. It remains a mystery if those cells came from BM or have a prenatal origin.

In summary, although EMH has been observed under both homeostatic and emergency conditions, it remains uncertain whether extramedullary precursors are remnants from early ontogeny or if they come from and are constantly replenished from the BM (Figure 2). Consequently, we wish to highlight the importance of thoroughly researching all mechanisms involved in these phenomena.

## 4 Molecules controlling hematopoiesis

The HSCs in the BM require a special microenvironment (niche) to maintain stemness. In the BM, two types of niches have been identified. First, the endosteal niche located in the inner cellular lining within bone cavity in contact with BM of trabecular bone, which is called endosteum. This region comprises specialized osteoblasts, CXCL12-abundant reticular cells (CARs), osteoclasts, and mesenchymal stromal cells (MSCs). Secondly, we have the vascular niche where HSCs are found next to CARs adjacent to BM sinusoids (134).

The molecules secreted from niche cells promote HSC maintenance. These include stem cell factor (SCF, KL) (135–136), CXCL12 (137, 138), angiopoietin 1 (139), and thrombopoietin (TPO) (140). Further detailed analysis of the BM niche showed that approximately 80% of dividing and non-dividing hematopoietic progenitors reside in close proximity to a sinusoid and nearly all HSCs are in contact with leptin receptor and CXCL12 high expressing niche cells (LEPR^+^CXCL12^*hi*^). The rest of the BM hematopoietic progenitors are located near arterioles (10%) and transition zones (10%) with only a few HSCs residing adjacent to the endosteum (141). In general, it is well-established that BM HSCs primarily inhabit these perivascular environments.

Other molecules are produced by a wide variety of cells that are essential for the regulation of hematopoiesis such as: SCF, notch ligands, bone morphogenic protein, transforming growth factor *β* (TGF-*β*), TPO, fibroblast growth factors, and insulin-like growth factor 2 as reviewed by (142). These molecules are necessary for maintaining and activating the resident HSCs on demand.

On the other hand, HSC and progenitor proliferation and differentiation is primarily regulated by cytokines like erythropoietin, granulocyte-monocyte colony-stimulating factor (GM-CSF) (143), *α*-interferon (INF-*α*) (144), and *γ*-interferon (INF- *γ*) (145). Hematopoiesis activated by inflammatory signals is often referred to as “emergency hematopoiesis” and it has been widely studied in pathogen associated inflammation (146).

Furthermore, factors produced during inflammatory processes in the mouse stimulate hematopoietic progenitors, some Toll-like receptor (TLR) ligands like Pam_3_CSK_4_ and lipopolysaccharide (LPS) promotes myelopoiesis *in vitro* (147). IFN-*γ* promotes activation and proliferation of HSCs both *in vitro* and *in vivo* (145). TLR3 ligand, poly (I:C), and its downstream effector INF-*α* also promotes the proliferation of dormant HSCs *in vivo* but chronic treatment caused the opposite effect (144). Other pro-inflammatory cytokines like G-CSF, interleukin (IL)-6, IL-11, and IL-12 promote not only HSC proliferation and differentiation but also HSC mobilization from BM to peripheral blood (148). The mobilization of HSCs is a mechanism by which sites of EMH may be seeded with hematopoietic precursors.

There are multiple cell sources of hematopoietic growth factors beyond innate immune cells that may contribute to EMH. In addition to the immune response, various cell types assist in the restoration and replenishment of hematopoietic niches. *In vitro* experiments involving the cultivation of HSCs with endothelial cells (ECs) or their soluble products have demonstrated that factors secreted by ECs are essential for enhancing the numbers of CFU-c (149). These factors are only partly defined. It was described that ECs are the main source of G-CSF in BM during inflammation, a molecule known to promote myelopoiesis (150). ECs also secrete IFN-*γ* and TNF-*α*, which promote MSC production of IL-13, another hematopoietic regulator in mice (151, 152). BM derived MSCs are affected by infection and inflammation, they respond to cytotoxic CD8^+^ T effector-cell signals and produce high levels of IL-6, thus promoting myelopoiesis (153). BM-MSCs primed by IFN-*γ* secretes hepatocyte growth factor, prostaglandin E2, that are known by its immune-suppressive effect but also stimulates growth of hematopoietic progenitors (154, 155). While BM niche molecules are known and widely described, our understanding of hematopoietic regulation outside the BM remains less understood.

## 5 EHM regulation by soluble factors

Hematopoietic progenitors respond to multiple signals, both in steady state and during inflammation, some of these signals are secreted molecules derived by numerous cell types throughout life as described before (156). Furthermore, the same pro-inflammatory molecules that promotes blood progenitor growth and mobilization in BM are linked to EMH in adults, such as GM-CSF, G-CSF, IL-3, IL-6 and IFN-*γ* (87, 157). Unfortunately, it is unknown which molecules outside the BM niche are specifically regulating the hematopoietic progenitors found in the extramedullary sites. While there are a number of potential candidates, some of them are discussed in more detail below:

### 5.1 Stromal derived factor 1

Certain soluble factors exhibit their function not only within the BM but also in extramedullary organs. For instance, stromal derived factor 1 (SDF-1), also recognized as CXCL12, is one such factor. Liver ECs sustain hematopoietic precursors both *in vitro* and *in vivo* by secreting SDF-1 and expressing adhesion molecules like CD34 similar to yolk sac endothelium capable of supporting hematopoietic proliferation and differentiation (129, 158).

During periods of stress, it is observed that blood progenitors migrate from the bone marrow to extramedullary tissues. In the spleen of mice, perisinusoidal ECs and tcf21^+^ stromal cells are found in the red pulp, where they produce SCF and SDF-1 (159). Moreover, it has been shown that a humanized mouse model injected with adenovectors expressing human SDF-1 promoted mobilization of HSCs into the spleen and peripheral blood, leading to an increased number of circulating human CD41^+^ cells (160). In addition, human spleens exhibiting extramedullary hematopoiesis showed significantly higher expression of SDF-1 compared to EMH-negative cases (161).

### 5.2 Thymic stromal lymphopoietin

Another possible EMH mediator is the thymic stromal lymphopoietin (TSLP). TSLP is a cytokine, that plays a crucial role in the regulation of the immune system and inflammation (162). TSLP is primarily produced at barrier surfaces in the body, such as the, gut, and lungs (163). Moreover, TSLP is involved in initiating and modulating immune responses at these barrier sites (164).

TSLP receptor is expressed by BM precursors and CD34^+^ cells in the periphery, the administration of exogenous TSLP to mice increased the percentages and numbers of Lin^−^CD34^+^ckit^+^ cells in the spleen, TSLP is mostly produced by epithelial and stromal cells and is involved in type 2 cytokine-mediated inflammation (165).

### 5.3 Isthmin-1

The regulation of extramedullary hematopoiesis (EMH) remains poorly understood, especially during homeostasis. While stress conditions offer some insights, questions persist about the origin and presence of resident blood precursors in extramedullary organs during steady-state conditions. As mentioned before, murine lungs contain fully functional hematopoietic progenitors (116). Nonetheless, a comprehensive characterization of the hematopoietic niche in the lungs has yet to be achieved, and certain researchers speculate that pericytes and ECs could potentially serve as niches for hematopoietic cells within the lung (126). Isthmin-1 is expressed by lung progenitors cells that are of hematopoietic and endothelial origin in the mouse (166). This soluble protein is related to NODAL subfamily of TGF-*β*, thus regulating embryonic development (167, 168). Additionally, Isthmin-1 plays a signaling role in promoting the development and functionality of hematopoietic tissues in the embryonic zebrafish (169). Hence, Ishtmin-1 has the potential to influence the formation of the hematopoietic microenvironment in other species and possibly outside the BM.

### 5.4 Adrenomedullin

Adrenomedullin (ADM) is a peptide hormone that plays a crucial role in regulating various physiological processes in the human body. It was first discovered in the adrenal medulla, which is why it is named “adrenomedullin” (170). This peptide has shown to improve *in vitro* expansion of cord blood HSCs in addition to classic cytokines such as SCF, TPO, EPO, FL3, GM-CSF, and GCSF (171, 172). Moreover, it has been demonstrated that ADM, of endothelial origin, is capable of maintaining HSCs in combination with SCF, TPO, and FLT3 ligand *in vitro* (173). Additionally, some primary BM stromal cell lines with the molecular signature Lin^−^CD45^−^CD271^+^PDGFRa^*low*/−^ also express ADM and are able to support human cord blood CD34^+^ cells (174).

While there is no conclusive evidence connecting ADM to the extramedullary support of hematopoietic progenitors, it is plausible that this peptide plays a role in EMH, given its widespread presence in organs such as the kidneys (175), heart (176), lungs (177), and blood vessels (173, 178).

### 5.5 Glial-derived neurotrophic factor

Another potential regulator of hematopoietic progenitors is the glial cell-derived neurotrophic factor (GDNF). GDNF, a member of the neurotrophic factor family, is a protein pivotal for the development, upkeep, and viability of numerous nerve cell types, particularly within the peripheral and central nervous systems (179). Recently, is has been shown that GNDF regulate hematopoiesis via RET signaling. Grey et al. demonstrated that the glial family receptor, RET, present on the surface of HSCs, contributes to prolonged cellular growth, increased stress resilience, and enhanced cell survival during *in vitro* expansion (180). When HSCs are exposed to RET ligand, GNDF, and its coreceptor complex, they exhibit enhanced progenitor function during primary transplantation and improved long-term HSC function during secondary transplantation. Human umbilical cord MSCs, when isolated, have the capacity to generate and release GDNF, contributing to tissue repair. Initially focused on nerve repair, this capability now extends to potentially include hematopoietic cells (181). Interestingly, murine BM HSCs also express RET and respond to it signaling partners, including GDNF (182). RET is not only expressed by BM cells but also for gut-associated lymphoid tissue, its signaling is necessary for Peyer’s patch formation (183). Generally, GDNF production and function is widely described within communication between neurons and their targets both in central as well as peripheral nervous system (184). It is noteworthy that GDNF can also be synthesized by MSCs and influence other types of cells such as salivary stem cells (185) or endothelial cells (186). It is conceivable that GDNF may exert an influence on hematopoietic precursors outside the bone marrow, analogous to its role in intestinal organogenesis.

## 6 Conclusions

In summary, the significance of extramedullary hematopoiesis (EMH) extends from embryonic development to adulthood, showcasing the dynamic changes in hematopoietic locations. Throughout development, the regulation of hematopoiesis by crucial cell types and molecules shapes the transition from EMH to bone marrow hematopoiesis. Some of these developmental EMH sites may maintain conditions conducive to hosting primitive and definitive hematopoietic progenitors. Recent research revealed that hematopoiesis originating from embryonic hemogenic ECs continues into adulthood, as evidenced even in 12-month-old mice (187). Notably, this research demonstrated that the majority of MPPs, T cells, and B-2 cells within the first month after birth were fetal EC-derived. Whether a similar phenomenon occurs in humans remains to be elucidated. Thus, understanding the origin of HSPCs as well as the variances in niches between normal and aberrant sites is vital, as it influences our comprehension of hematopoietic cell replenishment mechanisms. While BM niche is extensively characterized, with knowledge about cellular components and soluble factors, much remains unknown about the factors influencing adult EMH sites.

Most stem cell niches are situated near blood vessels and stromal cells (188–190). HSCs are no exception (191). Considering the nature of EMH sites, it is highly likely that the cells supporting these hematopoietic precursors are primarily endothelial and stromal cells. There is evidence showing that the vascular fraction of murine extramedullary adult organs like brain, heart, lung and liver are able to maintain LSK cells *in vitro* (192). In this context, the reliance on endothelial and stromal cells within EMH sites underscores the pivotal role of these cellular components in sustaining hematopoietic potential beyond the bone marrow.

## 7 Future perspectives

Nevertheless, there exist numerous inquiries that require further resolution. Are these possible niches similar to one another? What distinctions exist between the BM vascular niche and the vascular fraction of the organs displaying typical EMH? Additional research is needed to uncover the mechanisms that regulate the EMH niche-like functions. Undoubtedly, it is crucial to assess and contrast the various cell types participating in EMH with those of the perivascular and/or endosteal cells found in the BM niche.

The more recent models for investigating native hematopoiesis, exemplified by studies such as (193), hold promise for significant advancements in our understanding of hematopoietic processes. These models often incorporate advanced technologies, such as single-cell sequencing and sophisticated imaging techniques, allowing for a finer resolution of cellular interactions and dynamics within the hematopoietic microenvironment.

For instance, in a study where a high-resolution characterization of the bone marrow (BM) niche was conducted in both healthy conditions and acute myeloid leukemia (AML), researchers established a singlecell gene expression database encompassing 339,381 BM cells. The findings revealed significant alterations in cell type proportions and gene expression in AML, suggesting a comprehensive disruption of the entire niche (194). Such high-resolution analyses have the potential to enhance our comprehension of the pathological and homeostatic distinctions within extramedullary niches, spanning from development to adulthood.

## Figures and Tables

**TABLE 1:** Human adult EMH in disease.

Main organ or tissue affected	Associated disease (s)	Diagnostic technique	Reference
Brain hematoma	Congenital anomaly and anemia	Biopsy and pathological examination with IHC	-90
Brain hematoma	Unknown	CT Scan, biopsy, and pathological examination	-91
Brain mass	Thalassemia	MRI and pathological examination	-92
Brain	Myelofibrosis	CT Scan, MRI, and Tc99m sulfur colloid scan	-93
Spinal cord	Unknown	CT-guided biopsy, peripheral blood smears, BM aspirate, and pathological examination	-94
Spinal cord	Congenital anomaly	MRI, CT scan, biopsy, and pathological examination	-95
Spinal cord	Breast cancer	MRI, biopsy, and pathological examination	-96
Spinal cord	Thalassemia	MRI	-97
Thorax	Thalassemia	X-ray, MRI, biopsy and pathological examination and FC	-98
Thorax	Thalassemia	X-ray, CT scan, biopsy and pathological examination with IHC	-99
Lungs	Myelodysplasia and cirrhosis	X-ray and CT scan	-100
Lungs	Myeloproliferative disease and BM fibrosis	X-ray, CT scan, aspirate of pulmonary artery, and pathological examination	-101
Lungs	Sickle cell trait/β thalassemia	X-ray and autopsy findings	-102
Lungs	Myelofibrosis with myeloid metaplasia	X-ray, CT scan, blood smear, FC and Tc99m-anti-CD66 lung uptake	-103
Lungs	Myelofibrosis	CT99m Tc-anti-CD66, CT scan, biopsy and pathological examination with IHC	-104
Liver	Myeloid metaplasia	CT99m Tc-anti-CD66, CT scan, Tc99m sulfur colloid scan and biopsy with pathological examination	-105
Liver	Renal cancer	CT99m Tc-anti-CD66, X-ray, CT scan, and biopsy with pathological examination and IHC	-106
Liver	Myelofibrosis	CT99m Tc-anti-CD66, CT scan, biopsy, and BM smear with pathological examination	-107
Liver	Myeloid metaplasia	CT99m Tc-anti-CD66, CT scan, BM smear, liver mass aspirate, and pathological examination	-108
Liver	Lupus erythematosus	CT99m Tc-anti-CD66, CT scan, BM smear, liver biopsy, and pathological examination	-109
Spleen	Metastatic carcinoma	CT99m Tc-anti-CD66, sections of spleen and BM	-110
Spleen	Multiple myeloma	CT99m Tc-anti-CD66, Splenectomy and pathological examination with IHC	-111
Spleen	Gray platelet syndrome with myelofibrosis	CT99m Tc-anti-CD66, BM and blood smear, splenectomy and pathological examination	-112
Spleen & liver	Fibrous dysplasia	CT99m, Tc-anti-CD66, AP film, BM from surgical waste, liver biopsy, splenectomy, pathological examination	-113
Spleen & liver	Myelodysplastic syndromes	CT99m Tc-anti-CD66, imaging studies and biopsies	-114
